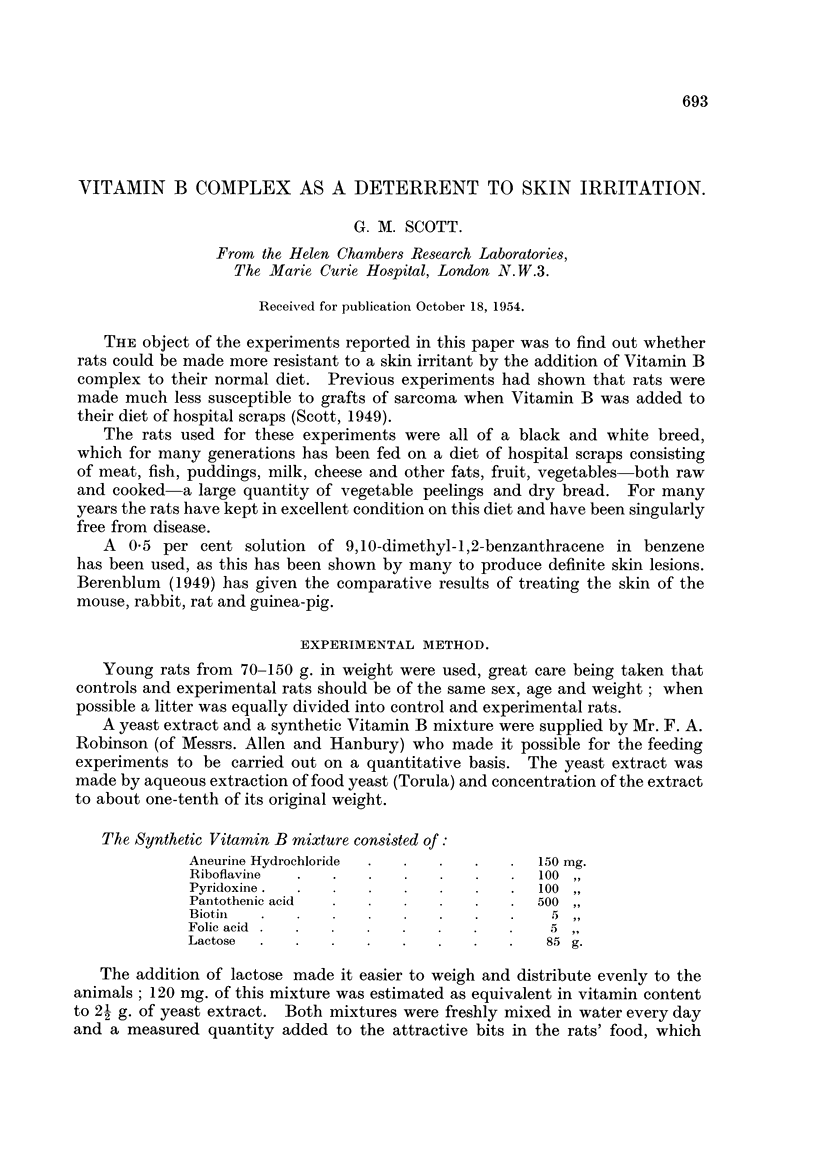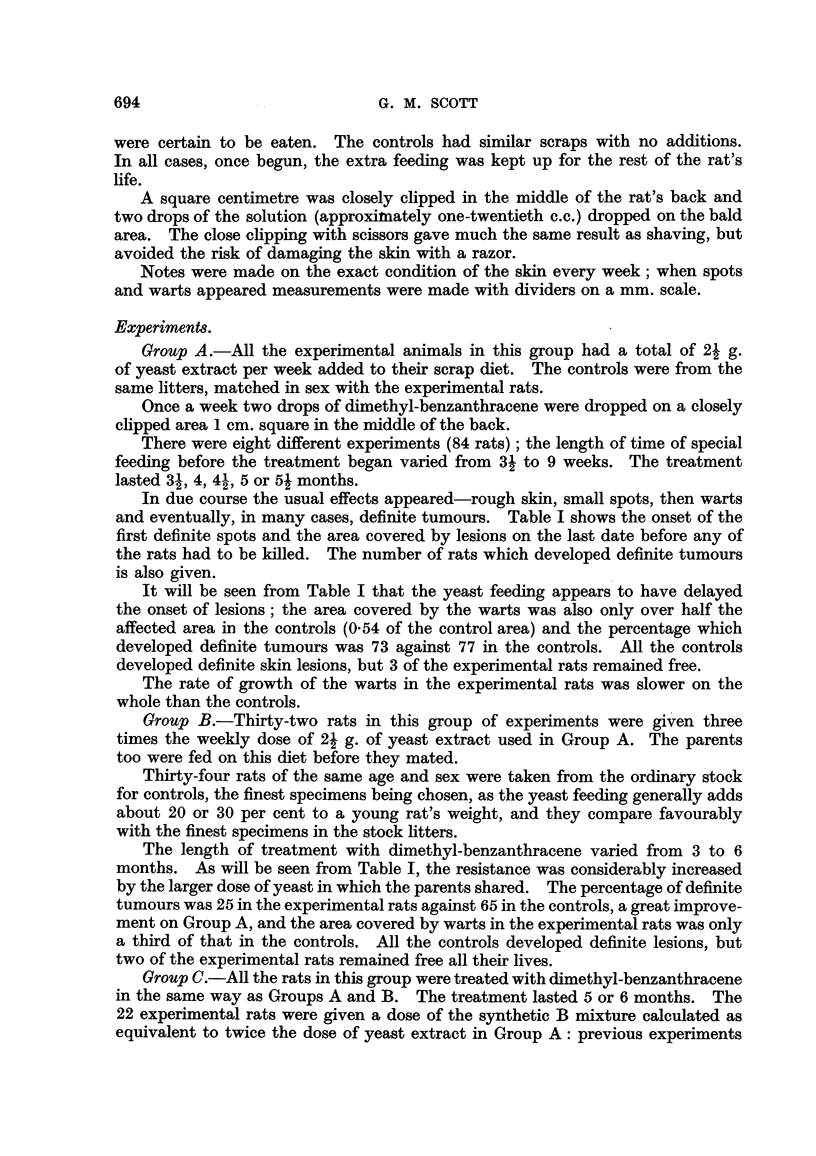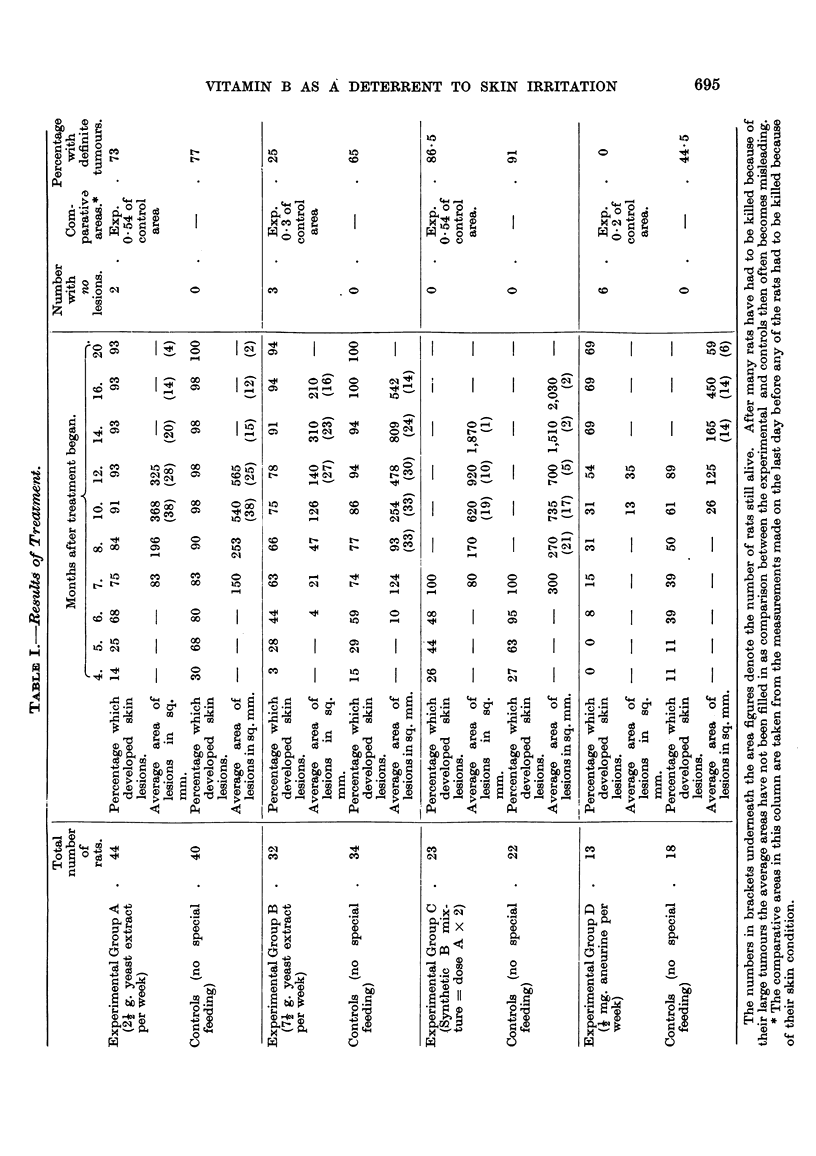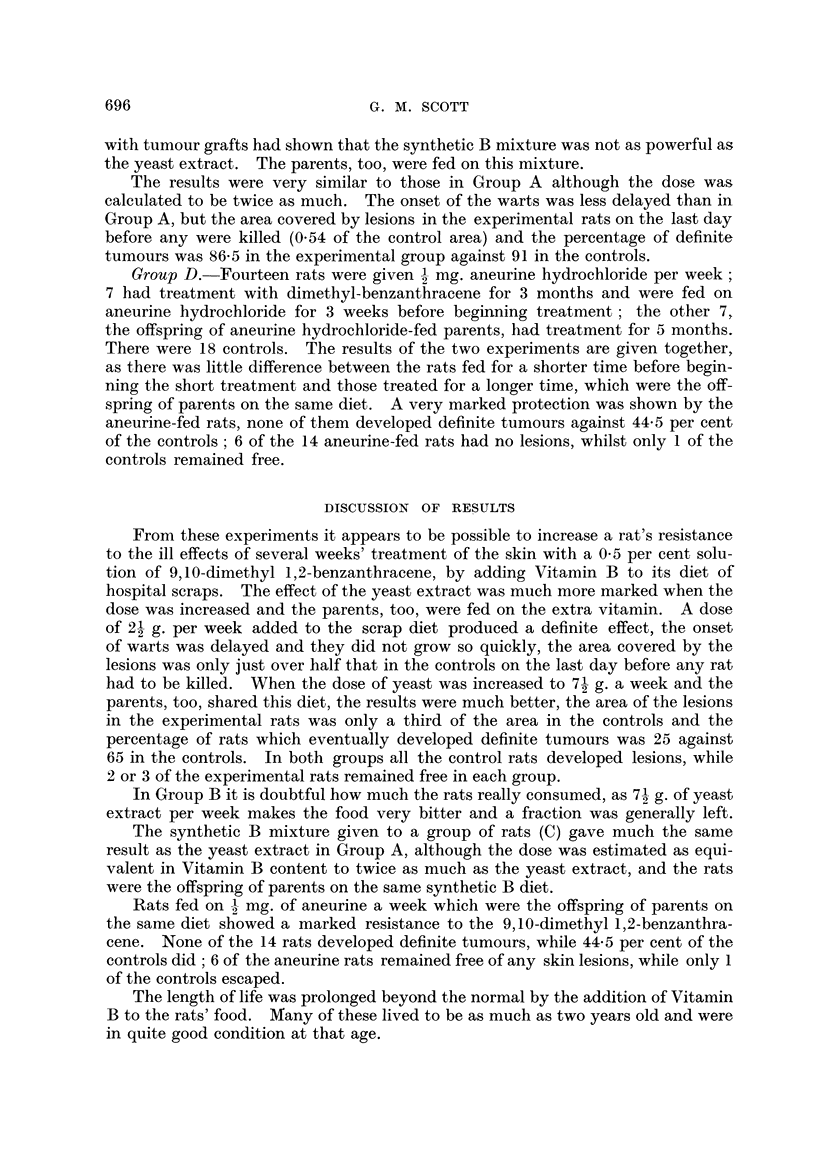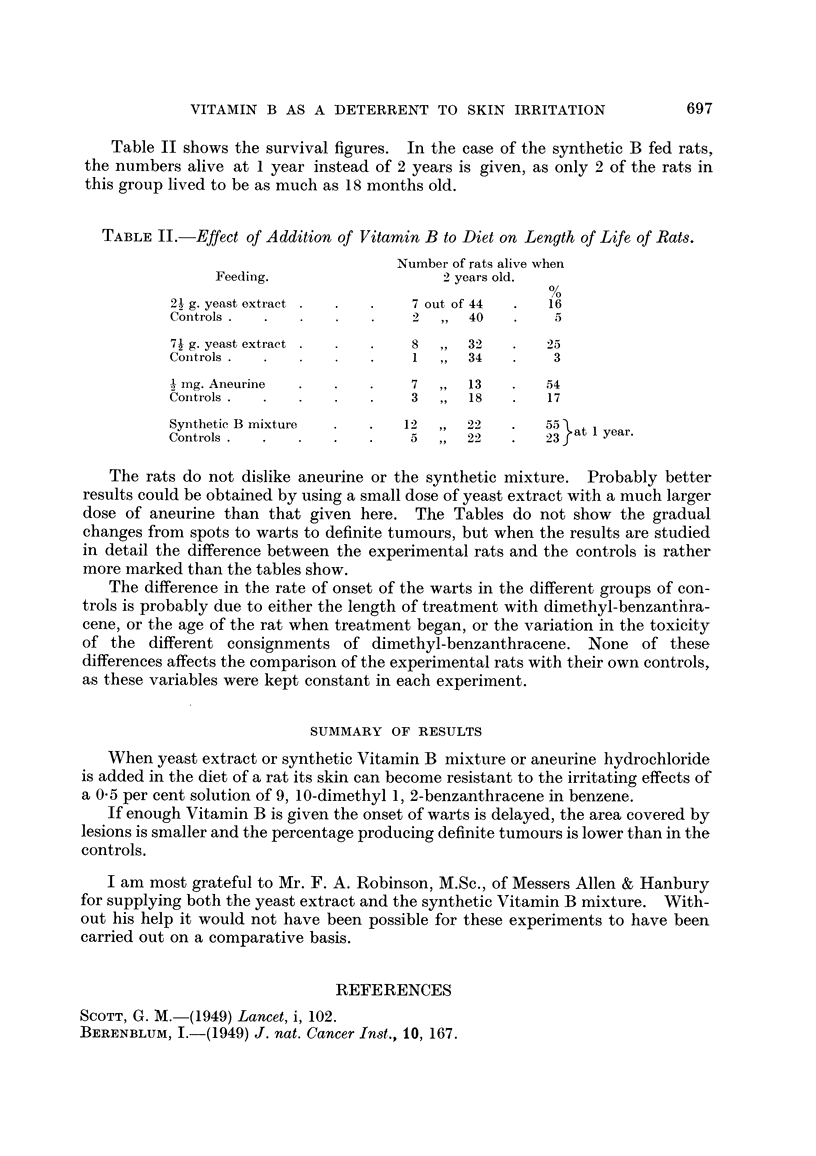# Vitamin B Complex as a Deterrent to Skin Irritation

**DOI:** 10.1038/bjc.1954.75

**Published:** 1954-12

**Authors:** G. M. Scott


### VITAMIN B COMPLEX AS A DETERRENT TO SKIN IRRITATION.

G. M. SCOTT.

*From the Helen Chambers Research Laboratories,*
*The Marie Curie Hospital, London N.W.3.*

Received for publication October 18, 1954.

THE object of the experiments reported in this paper was to find out whether rats could be made more resistant to a skin irritant by the addition of Vitamin B complex to their normal diet. Previous experiments had shown that rats were made much less susceptible to grafts of sarcoma when Vitamin B was added to their diet of hospital scraps (Scott, 1949).

The rats used for these experiments were all of a black and white breed, which for many generations has been fed on a diet of hospital scraps consisting of meat, fish, puddings, milk, cheese and other fats, fruit, vegetables—both raw and cooked—a large quantity of vegetable peelings and dry bread. For many years the rats have kept in excellent condition on this diet and have been singularly free from disease.

A 0·5 per cent solution of 9,10-dimethyl-1,2-benzanthracene in benzene has been used, as this has been shown by many to produce definite skin lesions. Berenblum (1949) has given the comparative results of treating the skin of the mouse, rabbit, rat and guinea-pig.

### EXPERIMENTAL METHOD.

Young rats from 70–150 g. in weight were used, great care being taken that controls and experimental rats should be of the same sex, age and weight ; when possible a litter was equally divided into control and experimental rats.

A yeast extract and a synthetic Vitamin B mixture were supplied by Mr. F. A. Robinson (of Messrs. Allen and Hanbury) who made it possible for the feeding experiments to be carried out on a quantitative basis. The yeast extract was made by aqueous extraction of food yeast (Torula) and concentration of the extract to about one-tenth of its original weight.

*The Synthetic Vitamin B mixture consisted of :*

| | |
|---|---|
| Aneurine Hydrochloride . . . . . . | 150 mg. |
| Riboflavine . . . . . . . . . | 100 „ |
| Pyridoxine . . . . . . . . . | 100 „ |
| Pantothenic acid . . . . . . . | 500 „ |
| Biotin . . . . . . . . . . | 5 „ |
| Folic acid . . . . . . . . . | 5 „ |
| Lactose . . . . . . . . . . | 85 g. |

The addition of lactose made it easier to weigh and distribute evenly to the animals ; 120 mg. of this mixture was estimated as equivalent in vitamin content to $2\frac{1}{2}$ g. of yeast extract. Both mixtures were freshly mixed in water every day and a measured quantity added to the attractive bits in the rats' food, which were certain to be eaten. The controls had similar scraps with no additions. In all cases, once begun, the extra feeding was kept up for the rest of the rat's life.

A square centimetre was closely clipped in the middle of the rat's back and two drops of the solution (approximately one-twentieth c.c.) dropped on the bald area. The close clipping with scissors gave much the same result as shaving, but avoided the risk of damaging the skin with a razor.

Notes were made on the exact condition of the skin every week; when spots and warts appeared measurements were made with dividers on a mm. scale.

*Experiments.*

*Group A.*—All the experimental animals in this group had a total of 2½ g. of yeast extract per week added to their scrap diet. The controls were from the same litters, matched in sex with the experimental rats.

Once a week two drops of dimethyl-benzanthracene were dropped on a closely clipped area 1 cm. square in the middle of the back.

There were eight different experiments (84 rats); the length of time of special feeding before the treatment began varied from 3½ to 9 weeks. The treatment lasted 3½, 4, 4½, 5 or 5½ months.

In due course the usual effects appeared—rough skin, small spots, then warts and eventually, in many cases, definite tumours. Table I shows the onset of the first definite spots and the area covered by lesions on the last date before any of the rats had to be killed. The number of rats which developed definite tumours is also given.

It will be seen from Table I that the yeast feeding appears to have delayed the onset of lesions; the area covered by the warts was also only over half the affected area in the controls (0·54 of the control area) and the percentage which developed definite tumours was 73 against 77 in the controls. All the controls developed definite skin lesions, but 3 of the experimental rats remained free.

The rate of growth of the warts in the experimental rats was slower on the whole than the controls.

*Group B.*—Thirty-two rats in this group of experiments were given three times the weekly dose of 2½ g. of yeast extract used in Group A. The parents too were fed on this diet before they mated.

Thirty-four rats of the same age and sex were taken from the ordinary stock for controls, the finest specimens being chosen, as the yeast feeding generally adds about 20 or 30 per cent to a young rat's weight, and they compare favourably with the finest specimens in the stock litters.

The length of treatment with dimethyl-benzanthracene varied from 3 to 6 months. As will be seen from Table I, the resistance was considerably increased by the larger dose of yeast in which the parents shared. The percentage of definite tumours was 25 in the experimental rats against 65 in the controls, a great improvement on Group A, and the area covered by warts in the experimental rats was only a third of that in the controls. All the controls developed definite lesions, but two of the experimental rats remained free all their lives.

*Group C.*—All the rats in this group were treated with dimethyl-benzanthracene in the same way as Groups A and B. The treatment lasted 5 or 6 months. The 22 experimental rats were given a dose of the synthetic B mixture calculated as equivalent to twice the dose of yeast extract in Group A: previous experiments with tumour grafts had shown that the synthetic B mixture was not as powerful as the yeast extract. The parents, too, were fed on this mixture.

The results were very similar to those in Group A although the dose was calculated to be twice as much. The onset of the warts was less delayed than in Group A, but the area covered by lesions in the experimental rats on the last day before any were killed (0·54 of the control area) and the percentage of definite tumours was 86·5 in the experimental group against 91 in the controls.

*Group D.*—Fourteen rats were given $\frac{1}{2}$ mg. aneurine hydrochloride per week ; 7 had treatment with dimethyl-benzanthracene for 3 months and were fed on aneurine hydrochloride for 3 weeks before beginning treatment ; the other 7, the offspring of aneurine hydrochloride-fed parents, had treatment for 5 months. There were 18 controls. The results of the two experiments are given together, as there was little difference between the rats fed for a shorter time before beginning the short treatment and those treated for a longer time, which were the offspring of parents on the same diet. A very marked protection was shown by the aneurine-fed rats, none of them developed definite tumours against 44·5 per cent of the controls ; 6 of the 14 aneurine-fed rats had no lesions, whilst only 1 of the controls remained free.

### DISCUSSION OF RESULTS

From these experiments it appears to be possible to increase a rat's resistance to the ill effects of several weeks' treatment of the skin with a 0·5 per cent solution of 9,10-dimethyl 1,2-benzanthracene, by adding Vitamin B to its diet of hospital scraps. The effect of the yeast extract was much more marked when the dose was increased and the parents, too, were fed on the extra vitamin. A dose of $2\frac{1}{2}$ g. per week added to the scrap diet produced a definite effect, the onset of warts was delayed and they did not grow so quickly, the area covered by the lesions was only just over half that in the controls on the last day before any rat had to be killed. When the dose of yeast was increased to $7\frac{1}{2}$ g. a week and the parents, too, shared this diet, the results were much better, the area of the lesions in the experimental rats was only a third of the area in the controls and the percentage of rats which eventually developed definite tumours was 25 against 65 in the controls. In both groups all the control rats developed lesions, while 2 or 3 of the experimental rats remained free in each group.

In Group B it is doubtful how much the rats really consumed, as $7\frac{1}{2}$ g. of yeast extract per week makes the food very bitter and a fraction was generally left.

The synthetic B mixture given to a group of rats (C) gave much the same result as the yeast extract in Group A, although the dose was estimated as equivalent in Vitamin B content to twice as much as the yeast extract, and the rats were the offspring of parents on the same synthetic B diet.

Rats fed on $\frac{1}{2}$ mg. of aneurine a week which were the offspring of parents on the same diet showed a marked resistance to the 9,10-dimethyl 1,2-benzanthracene. None of the 14 rats developed definite tumours, while 44·5 per cent of the controls did ; 6 of the aneurine rats remained free of any skin lesions, while only 1 of the controls escaped.

The length of life was prolonged beyond the normal by the addition of Vitamin B to the rats' food. Many of these lived to be as much as two years old and were in quite good condition at that age.

Table II shows the survival figures. In the case of the synthetic B fed rats, the numbers alive at 1 year instead of 2 years is given, as only 2 of the rats in this group lived to be as much as 18 months old.

TABLE II.—*Effect of Addition of Vitamin B to Diet on Length of Life of Rats.*

| Feeding. | Number of rats alive when 2 years old. | % | |
|---|---|---|---|
| $2\frac{1}{2}$ g. yeast extract | 7 out of 44 | 16 | |
| Controls | 2 ,, 40 | 5 | |
| $7\frac{1}{2}$ g. yeast extract | 8 ,, 32 | 25 | |
| Controls | 1 ,, 34 | 3 | |
| $\frac{1}{2}$ mg. Aneurine | 7 ,, 13 | 54 | |
| Controls | 3 ,, 18 | 17 | |
| Synthetic B mixture | 12 ,, 22 | 55 | at 1 year. |
| Controls | 5 ,, 22 | 23 | at 1 year. |

The rats do not dislike aneurine or the synthetic mixture. Probably better results could be obtained by using a small dose of yeast extract with a much larger dose of aneurine than that given here. The Tables do not show the gradual changes from spots to warts to definite tumours, but when the results are studied in detail the difference between the experimental rats and the controls is rather more marked than the tables show.

The difference in the rate of onset of the warts in the different groups of controls is probably due to either the length of treatment with dimethyl-benzanthracene, or the age of the rat when treatment began, or the variation in the toxicity of the different consignments of dimethyl-benzanthracene. None of these differences affects the comparison of the experimental rats with their own controls, as these variables were kept constant in each experiment.

### SUMMARY OF RESULTS

When yeast extract or synthetic Vitamin B mixture or aneurine hydrochloride is added in the diet of a rat its skin can become resistant to the irritating effects of a 0·5 per cent solution of 9, 10-dimethyl 1, 2-benzanthracene in benzene.

If enough Vitamin B is given the onset of warts is delayed, the area covered by lesions is smaller and the percentage producing definite tumours is lower than in the controls.

I am most grateful to Mr. F. A. Robinson, M.Sc., of Messers Allen & Hanbury for supplying both the yeast extract and the synthetic Vitamin B mixture. Without his help it would not have been possible for these experiments to have been carried out on a comparative basis.

### REFERENCES

SCOTT, G. M.—(1949) *Lancet*, i, 102.

BERENBLUM, I.—(1949) *J. nat. Cancer Inst.*, **10**, 167.